# The Cell Wall Sensors Mtl1, Wsc1, and Mid2 Are Required for Stress-Induced Nuclear to Cytoplasmic Translocation of Cyclin C and Programmed Cell Death in Yeast

**DOI:** 10.1155/2013/320823

**Published:** 2013-10-23

**Authors:** Chunyan Jin, Andrey V. Parshin, Ira Daly, Randy Strich, Katrina F. Cooper

**Affiliations:** ^1^Department of Molecular Biology, Graduate School of Biomedical Sciences, Rowan University, Stratford, NJ 08055, USA; ^2^Department of Cell Biology, Graduate School of Biomedical Sciences, Rowan University, Stratford, NJ 08055, USA; ^3^Quintiles Transnational Corp., Morris Plains, NI 07950, USA; ^4^Department of Molecular Biology, Graduate School of Biomedical Sciences, Rowan University, Two Medical Center Drive, Stratford, NJ 08084, USA

## Abstract

Mtl1 is a member of a cell wall sensor family that monitors cell wall integrity in budding yeast. In response to cell wall stress, Mtl1 activates the cell wall integrity (CWI) MAP kinase pathway which transmits this signal to the nucleus to effect changes in gene expression. One target of the CWI MAP kinase is cyclin C, a negative regulator of stress response genes. CWI activation results in cyclin C relocalization from the nucleus to the cytoplasm where it stimulates programmed cell death (PCD) before it is destroyed. This report demonstrates that under low oxidative stress conditions, a combination of membrane sensors, Mtl1 and either Wsc1 or Mid2, are required jointly to transmit the oxidative stress signal to initiate cyclin C destruction. However, when exposed to elevated oxidative stress, additional pathways independent of these three sensor proteins are activated to destroy cyclin C. In addition, *N*-glycosylation is important for Mtl1 function as mutating the receptor residue (Asn42) or an enzyme required for synthesis of *N*-acetylglucosamine (Gfa1) reduces sensor activity. Finally, combining *gfa1-1* with the cyclin C null allele induces a severe synthetic growth defect. This surprising result reveals a previously unknown genetic interaction between cyclin C and plasma membrane integrity.

## 1. Introduction

The yeast cell wall is the first level of defense against environmental stress. Embedded in this cell wall are sensors that detect damage and transduce this signal to the nucleus to change gene expression. In addition, this stress signal must then be disseminated throughout the cell to elicit changes in organelle function and morphology. For example, exposure to oxidative stress induces the transcription of protein chaperones and antioxidant enzymes, alters ribosome assembly in the nucleolus, and triggers extensive fragmentation of the mitochondria [1]. All three examples help inform the cell on deciding whether to arrest cell growth and repair the damage or execute the programmed cell death pathway.

**Figure 1:**
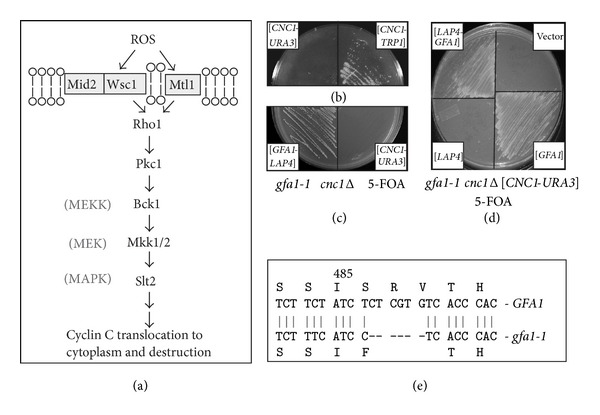
Negative synthetic growth defect in a *cnc1Δ gfa1-1 *mutant. (a) Model of the cell wall signaling pathway that regulates cyclin C cytoplasmic translocation and consequent degradation. Adapted from [5] and data presented in [6, 7, 25] and this report. (b) An isolate following EMS mutagenesis harboring either a *URA3* (left half) or *TRP1* (right half) marked *CNC1* expression plasmid was streaked on medium containing 5-FOA. (c) and (d) The *gfa1-1 cnc1Δ* double mutant strain was transformed with the indicated plasmids then streaked on 5-FOA medium. All plates were incubated for 3 days at 30° before the image was taken. (e) Sequence analysis of the *gfa1-1* allele is shown. The six nucleotide deletion is indicated by the hash marks. The predicted amino acid sequences for the wild type and *gfa1-1 *encoded proteins are indicated in single amino acid code.

In yeast, the cell wall integrity pathway (CWI) responds to a variety of stresses, including oxidative stress [2], heat shock [3], and any other stress that alters the cell wall integrity. In brief, the CWI pathway senses the stress via a family of cell-surface sensors (Wsc1, Mid2, and Mtl1 [4]). These sensors transmit the stress to a small G protein Rho1, which in turn activates protein kinase C (Pkc1). The activated Pkc1 transmits the intracellular signals to the MAPK Slt2/Mpk1 via the MAPK module (see Figure 1(a)). Finally, the transcription factors (Rlm1, Swi4/Swi6) regulating the corresponding gene expression act as the output of the CWI pathway (Figure 1(a) and reviewed in [5]). In addition, oxidative stress activation of this pathway triggers the Slt2-dependent cytoplasmic translocation and consequent degradation of cyclin C [6, 7].

The cell wall sensor proteins are members of either the Mid2 or Wsc subfamily [4]. The Wsc sensor subfamily includes Wsc1, the main Wsc senor [8], Wsc2, and Wsc3 [9]. The Mid2 subfamily contains Mid2 and Mtl1 that shares 50% homology with Mid2 [10]. Although these cell wall sensors share structural similarity, their sequences are not conserved. The Wsc subfamily contains an N-terminal cysteine-rich region, termed the CRD domain, which is not present in Mid2 or Mtl1. The CRD domain transiently interacts with the glucan chains in the cell wall, while the transmembrane domain anchors the sensor in the plasma membrane. In response to cell wall stress, the glucan chains are stretched, exerting a force on the nanospring-like Ser/Thr-rich domain. This results in a conformational change within the cytoplasmic domain, which triggers the interaction with Rom2 and activates the downstream signaling cascade (reviewed in [11]). Intermolecular interactions of the CRD domains promote sensor clustering, with a concomitant increase of the downstream signaling. This local accumulation enhances the stress signal and the cellular response [12]. 

Posttranslational modification is required for proper function of these cell wall sensors. The *N*-glycosylation of Asn35 is required for Mid2-dependent Slt2 activation [13]. However, this modification is not necessary for deposition of Mid2 to the plasma membrane suggesting that glycosylation is important for the protein to initiate the stress signal. The production of UDP-*N*-acetyl-D-glucosamine (UDP-GlcNAc), a building block for *N*- and *O-*linked glycosylation, as well as the formation of GPI-anchors and chitin (reviewed in [14]) is controlled by both extra- and intracellular cues. For example, *GFA1, *which encodes glutamine fructose-6-phosphate aminotransferase, an enzyme that catalyzes the first and rate-limiting step in UDP-GlcNAc production, is regulated in response to mating pheromones [15], cell cycle progression [16, 17], and cell wall damage [18]. Defects in this pathway result in hypersensitivity to cell wall damage induced by heat shock [19] or spore wall morphogenesis [20]. Defects in glycosylation also result in cell death [21, 22] via Kex1, a protease involved in programmed cell death induced by acetic acid or chronological aging [23]. These results suggest an intimate relationship between glycosylation, stress signaling, and the execution of cell death programs in yeast.

The mechanisms underlying transcription factor activation by signal transduction pathways have garnered much of the attention when studying how exogenous cues are converted into changes in the gene expression program [24]. The other side of the coin, that is, removal of repressor proteins, is not as well understood. In budding yeast, repression of many stress response genes (SRG) including the *HSP70* member *SSA1* [25], *CIT1,* and *DDR2* [6] is mediated by the cyclin C-cyclin-dependent kinase 8 (Cdk8) module [26, 27]. This complex associates with the mediator component of the RNA polymerase holoenzyme and plays both a positive and negative roles in transcription depending on the specific locus [28–30]. Unlike cyclins that regulate the cell cycle, cyclin C levels do not vary significantly during the cell cycle in yeast or human cells [25, 31]. To relieve cyclin C-Cdk8-dependent repression in yeast, cyclin C is destroyed by a Not4-dependent process in cultures subjected to a variety of stressors [6]. Before it is destroyed, cyclin C (but not Cdk8) translocates to the cytoplasm [6] where it is required for stress-induced mitochondrial hyperfission (unpublished data; K. F. Cooper, S. Khakhina, S. K. Kim, and R. Strich). Mitochondrial hyper-fission is a conserved hallmark of the stress response in higher eukaryotes [32–34] as well as yeast [35–37] (see [38] for review). In many examples, mitochondrial fission is an early event in the PCD pathway [39, 40]. Thus, the resistance to ROS-induced programmed cell death (PCD) exhibited by cyclin C null cells [6, 7] is likely due to a defect in the extensive mitochondrial fragmentation associated with cellular damage. These results indicate that the normal cellular response to oxidative stress requires the proper function of cell wall sensors that transduce the signal to the nucleus to mediate translocation of cyclin C to the cytoplasm. This study connects a complex sensor system requiring proper *N*-glycosylation through Gfa1 function to cyclin C relocalization, destruction, and programmed cell death.

## 2. Materials and Methods

### 2.1. Yeast Strains, Plasmids, and Cell Culturing Conditions

The strains used in this study are listed in Table 1 and most are derived from W303-related strains RSY10 (*MAT* **a** *ade2 ade6 can1-100 his3-11, 15 leu2-3, 112 trp1-1 ura3-1*) [41] or W303-1B [42]. In accordance with the Mediator nomenclature unification effort [43] cyclin C *(SSN8/UME3/SRB11)* and *Cdk8 (SSN3/UME5/SRB10)* we will use *CNC1* and *CDK8* gene designations, respectively. *KANMX4* deletion strains were constructed by integrating the PCR amplified *KANMX4* deleted alleles obtained from the Research Genetics deletion strain collection. Deletion alleles using other auxotrophic markers or the Mtl1-3HA and *pGAL-MTL1* strains were constructed using gene replacement methodology [44]. The *cnc1*Δ strains have been previously described [25]. All primers used are listed in Table 2. Details about the plasmids used in this study can be found in Table 3. The *CNC1* ORF was tagged with one copy of the myc epitope placed immediately downstream of initiator methionine and creates a functional protein [25]. The functional YFP-cyclin C fusion expression plasmid construct (pBK37) was made by replacing the GFP allele in the functional GFP-cyclin C construct, pBK1 [6] with PCR amplified YFP. pID301 was created in two steps. First, a 2.5 kbp *EcoR*1 fragment containing the *CNC1* minimal subclone [25] was cloned into *EcoR*1 digested pRS316 [45] to form pKC311. Second, *ADE2* was inserted into pKC311 by cloning the *BamH*I fragment from pAZ11 [46] into *Bgl*II cut pKC311 to form pID301. pJB323 was made by cloning the *CNC1* minimal subclone that contains its own promotor and terminator sequences into *ECOR1* cut pRS314 [45]. Further details are available upon request. The 3HA-tagged *MTL1* plasmids (pCJ3 and pCJ4) were made by PCR amplification using Phusion Taq (Denville Scientific) of the chromosomally tagged *MTL1* ORF, promotor and terminator from RSY1659. The PCR fragment was cut with *Sac*II and *EcoR*1 and the resulting fragment cloned into pRS423 or pRS426 [45], respectively, digested with the same enzymes. The *MTL1* ^*N42A*^ and *MTL1* ^*N547A*^ constructs were generated using site-directed mutagenesis on pCJ3 or pCJ4 according to the manufacturers direction (Stratagene) with oligonucleotides listed in Table 2. The remaining plasmids were gifts: pYO964 containing hyperactive *RHO1* allele (*G19V*) was provided by Y. Ohya. *BCK1-20* containing the constitutively active *BCK1* allele and the *SLT2*-HA expression plasmids were from D. Levin. Cells were grown in either rich, nonselective medium (YPDA) or synthetic minimal medium (SC) to allow for plasmid selection as previously described [25]. Galactose inducible gene expression was achieved by adding galactose (2% final concentration) to cultures grown in SC with raffinose as a carbon source.

### 2.2. Synthetic Lethality Screen

The colony color-sectoring method was used as previously described [47]. Strain (RSY397, *ade2 cnc1::LEU2 ura3*) harboring the *CNC1* gene on a plasmid with the *ADE2* and *URA3* selectable markers (pID301) was grown to midlog phase (8 × 10^6^), mutagenized with ethylmethane sulfonate (EMS) as previously described [41], and then plated on solid medium with limiting adenine (6 mg/mL). Colonies that were unable to lose pID301 (as demonstrated by an unsectored colony) were selected as candidates for further study. To further screen these candidates, the colonies were transferred to solid medium containing 5-fluoroorotic acid (5-FOA), an analog in the uracil biosynthetic pathway. Colonies able to lose the *CNC1* plasmid with the *URA3* gene would continue to grow. We identified thirteen colonies unable to lose the *CNC1* expression plasmid suggesting that *CNC1* was now required for growth. From this double screen, one individual (RSY1543) was chosen for further study as described in the text due to strength of the slow growth phenotype. Verification of the requirement for cyclin C was achieved by swapping pID301 for pJB323 and plating these cells on limiting adenine and 5-FOA medium. The mutant allele responsible for this synthetic phenotype was isolated by transforming RSY1543 harboring pID301 with the pRS200 (*TRP1-marked*) genomic library (ATCC 77164) and identifying cells that could grow on 5-FOA medium. Plasmid DNA (pKC800) was recovered by *E. coli* transformation from 5-FOA resistant cells that formed white colonies and retransformed back into RSY1543 to verify the complementation phenotype. pKC800 was sequenced and determined to contain two ORFS, *GFA1* and *LAP4*. pKC811 (*LAP4* ORF, promotor and terminator) and pKC812 (*GFA1* ORF promotor and terminator) were created from pKC800. 

### 2.3. Identification of the *gfa1-1* Mutation

The *gfa1-1 *mutation was identified by sequencing a PCR fragment generated from the chromosomal copy of the mutant allele in strain RSY1543. In brief, yeast DNA from RSY1543 was made as previously described [25] and amplified using KCO1234 and KCO1235 which map 200 bp upstream and 200 bp downstream of the *GFA1* ORF, respectively. This PCR fragment was then sequenced (Eurofins MWG Operon) and the results were aligned with wild type *GFA1* ORF. Further details of primers used for sequencing are available upon request.

### 2.4. Survival and Stress Assays

For all stress assays, cells were grown to midlog phase (6 × 10^6^ cells/mL) and then treated with H_2_O_2_ or acetic acid at the concentrations described in the text. Clonogenic viability studies were conducted with midlog phase (6 × 10^6^ cells/mL) treated with 100 mM acetic acid for 200 min and then serially diluted (1 : 10) and plated on minimal complete medium with or without plasmid selection as indicated in the text. Caspase assays were conducted with three independent cultures as described [48] except that the cells were incubated with the caspase substrate (CaspSCREEN BioVision Inc.) at 37° for 24 h in the dark. At least 20,000 cells were counted per sample. Statistical analysis was performed using the unpaired Student's *t*-test with *P* values <0.05 being considered significant. 

### 2.5. Western Blot Analysis

Extracts prepared for analyzing myc-cyclin C levels were prepared from midlog cultures (6 × 10^6^ cells/mL) as described previously [25] except that the lysis buffer used was 150 mM NaCl, 50 mM Tris-HCl pH 8.0, 1% NP-40, 0.15% deoxycholic acid sodium salt, 1 *μ*g/mL pepstatin, 1 *μ*g/mL leupeptin, and 0.2% protease inhibitor cocktail (Sigma). In brief, 500 *μ*g of soluble extract was immunoprecipitated using either anti-myc or anti-HA antibodies (Roche), collected on agarose A beads, and then analyzed by Western blot. For monitoring Mtl1 glycosylation, a gradient acrylamide gel was used (5–10%) to allow resolution of both modified and unmodified signals on the same gel. Western blot signals were detected using goat *α*-mouse secondary antibodies conjugated to alkaline phosphatase (Sigma) and the CDP-Star chemiluminescence kit (Tropix). Signals were quantitated by phosphorimaging (Kodak Inc.). Half-life determinations were calculated by linear regression analysis with curves possessing *r*  values >0.9. Relative cyclin C concentrations were standardized internally to Tub1 levels before comparing to other values. Slt2-HA phosphorylation was detected using *α*-phospho-p44/42 antibodies (Cell Signaling) as previously described [7]. Tub1 was visualized using *α*-tubulin antibodies (12G10) were obtained from the Developmental Studies Hybridoma Bank (University of Iowa).

### 2.6. N-Glycosylation Assay

Mtl1 *N-*glycosylation was monitored as described previously [13]. Briefly, crude membrane extracts were prepared from 250 mL midlog cultures harboring the Mtl1-3HA plasmid indicated (5 × 10^6^ cells/mL in minimal medium). An equal volume of glass beads to the cell pellet was added, and the cells lysed with 500 *μ*L lysis buffer (150 mM NaCl, 50 mM Tris-HCl pH 8.0, 1% NP-40, 0.15% deoxycholic acid sodium salt, 1 *μ*g/mL pepstatin, 1 *μ*g/mL leupeptin, and 0.2% protease inhibitor cocktail (Sigma)) by vortexing four times for 1 min (with 1 min intervals on ice). Cell debris was removed by centrifugation for 5 min at 3500 ×g at 4°C. Crude membranes were collected from the supernatant by centrifugation for 30 min at 18,000 ×g at 4°C (Beckman TLA-55 rotor) and resuspended in 100 *μ*L lysis buffer. The crude membranes were digested with 2,500 units of Endo H (New England Biolabs) for 2 h at 37°C. Mock incubations were carried out without Endo H. Reactions were stopped by adding 3X SDS sample buffer and analyzed by Western blot.

### 2.7. Immunofluorescence Microscopy

Localization studies of chimeric fusion proteins were performed on cells fixed in 3.7% paraformaldehyde and stained with 4′,6-diamidino-2-phenylindole (DAPI). For all experiments, the cells were grown to midlog (5 × 10^6^ cells/mL), treated with the H_2_O_2_ concentrations and timepoints indicated in the figures, and then analyzed by fluorescence microscopy. Images were obtained using a Nikon microscope (model E800) with a 60X objective (Plan Fluor Oil, NA 1.3) and a CCD camera (RETIGA Exi). Data were collected using NIS software and processed using Image Pro software. All images of individual cells were optically sectioned (0.2 *μ* slices at 0.6 *μ* spacing) and deconvolved. 

## 3. Results

### 3.1. Gfa1 and Cyclin C Display a Negative Genetic Interaction

Cyclin C is a target of the cell wall integrity pathway and required for programmed cell death [7]. To identify additional components of this regulatory network, a synthetic lethality screen was undertaken (see Section 2 for details). These studies identified a mutant that was unable to grow in the presence of the counterselection drug 5-FOA when cyclin C was expressed from a plasmid containing the *URA3* selectable marker (Figure 1(b), left panel). However, introduction of *CNC1* on a plasmid with the *TRP1* selectable marker was able to lose the *URA3* based plasmid indicating the cyclin C expression was required for normal cell growth (Figure 1(b), right panel). Continued incubation of these plates did permit limited growth of the double mutant strain (data not shown). This indicates that the phenotype observed was not due to synthetic lethality but rather a severe growth defect.

To identify the gene corresponding to the mutant allele responsible for this synthetic phenotype, a genomic library was introduced into this strain and transformants were identified that were now able to grow in the absence of cyclin C. One transformant was identified that contained a genomic contig with two intact genes, *GFA1* and *APE1/LAP4* (Figure 1(c), left panel). To determine which of these genes possessed the complementation activity, plasmids were introduced into the mutant strain expressing either *GFA1* or *APE1*. This experiment revealed that *GFA1* complemented the synthetic growth phenotype (Figure 1(d)). Since *GFA1* is an essential gene, our allele (*gfa1-1*) must be hypomorphic but still possessing sufficient activity for survival. To determine the nature of this allele, DNA sequence analysis was performed on PCR products specific to the *GFA1* coding region generated from the *gfa1-1* strain. This analysis revealed a six nucleotide deletion which changed S486 to a phenylalanine and deleted R487 and V488 (Figure 1(e)). The remainder of the protein appears unaltered. This small deletion resides in one of two sugar isomerase (SIS) domains found in many sugar binding proteins [49]. This result suggests that the small deletion in Gfa1 reduces, but does not eliminate, Gfa1 activity. 

### 3.2. Gfa1 Is Required for Cyclin C Destruction in Response to Oxidative Stress

**Figure 2:**
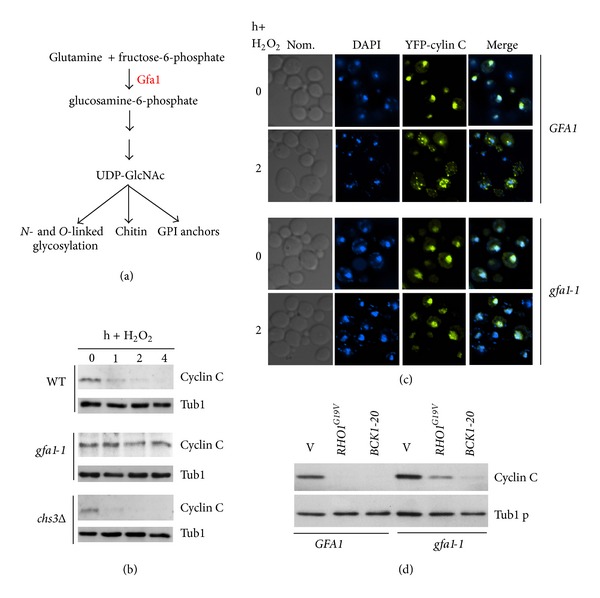
Gfa1 is required for cyclin C relocalization and destruction. (a) Diagram of Gfa1 functions in the cell. (b) Gfa1 is required for cyclin C destruction. Wild type (RSY10), *gfa1-1 cnc1Δ* (RSY1543), and *chs3Δ* (RSY1539) midlog phase cultures expressing myc-cyclin C (pLR337) were treated with 0.4 mM H_2_O_2_ for the indicated times in hours. Cyclin C levels were determined by Western blot analysis of immunoprecipitates. Tub1 levels were used as a loading control. (c) YFP-cyclin C subcellular localization was monitored in a wild type and *gfa1-1 cnc1Δ* strains harboring pBK37 before and following H_2_O_2_ treatment. DAPI staining was used to identify the nucleus. (d) Gfa1 functions upstream of the CWI pathway. Cyclin C levels were determined by Western blot analysis of immunoprecipitates in a midlog phase wild type (RSY10) or *gfa1-1 cnc1Δ* (RSY1543) cultures expressing myc-cyclin C (pLR337) and either a vector control (v), *BCK1-20* or *RHO1* ^G19V^. Tub1 levels were used as a loading control.

*GFA1* encodes an essential glutamine-fructose-6-phosphate aminotransferase that catalyzes the first step in GlcNAc biosynthesis [15]. GlcNAc is involved in several biological processes including GPI anchor formation, chitin biosynthesis, and the substrate for *N*- and *O*-linked glycosylation (Figure 2(a)). Given the established role of Gfa1 in cell wall maintenance and our previous findings that cell wall stressors induce cyclin C destruction, we next determined if Gfa1 is required for oxidative stress-induced degradation of cyclin C. A *CNC1* myc tagged allele under the control of the *ADH1* promoter was placed on a single copy plasmid and introduced into the *gfa1-1* mutant. Myc-cyclin C levels were monitored by Western blot analysis following exposure to hydrogen peroxide (0.4 mM). Compared to wild type, myc-cyclin C levels were not reduced in the *gfa1-1* strain (Figure 2(b)). These results indicate that Gfa1 is required for normal cyclin C destruction. However, chitin synthase (*CHS3*) is not required for cyclin C destruction (Figure 2(b)). Although other chitin synthases are present in the cell, *chs3* mutants display several phenotypes similar to *gfa1* mutants including spore wall assembly and temperature sensitive growth [50]. Taken together, these results suggest that Gfa1 functions other than stimulating chitin formation are involved in regulating cyclin C destruction.

Our previous report indicated that cyclin C relocalization from the nucleus to the cytoplasm was required for both cyclin C destruction and programmed cell death execution [6]. Therefore, we next asked whether Gfa1 was also required for cyclin C relocalization. A wild type and *gfa1-1* strain was transformed with a plasmid expressing YFP-cyclin C. We have previously demonstrated that this reporter protein was functional and recapitulated normal cyclin C regulation [6]. In response to H_2_O_2_ treatment, YFP-cyclin C foci were observed in the cytoplasm in the wild-type strain (Figure 2(c)). However, YFP-cyclin C remained predominantly nuclear in the *gfa1-1* strain. These results indicate that Gfa1 is required for ROS-induced nuclear to cytoplasmic translocation of cyclin C.

The results just described indicate that Gfa1 is required for the oxidative stress signal to induce cyclin C relocalization and destruction. We have reported that the cell wall integrity MAP kinase pathway is necessary for this process (see Figure 1(a) and [7]). To determine if Gfa1 functions upstream or downstream of this signaling pathway, epistasis experiments were conducted. Plasmids expressing either constitutively active alleles of *RHO1* (*RHO1* ^*G19V*^) or *BCK1* (*BCK1-20*) were introduced into a wild type strain and the *gfa1-1* mutant. Cyclin C levels were monitored in these cultures in the absence of stress. As previously reported [7] constitutive activation of Bck1 or Rho1 (this study) can induce cyclin C destruction in the absence of stress (Figure 2(d)). A similar result was obtained in the *gfa1-1 *strain. The low level retention of cyclin C in the *gfa1-1* culture expressing Rho1^G19V^ was reproducible suggesting that Gfa1 function is partially required for Rho1^G19V^-induced cyclin C destruction. These results indicate that Gfa1 mediates stress-induced cyclin C relocalization and destruction. These results suggest that Gfa1 may regulate cyclin C destruction through glycosylation of a CWI pathway component.

### 3.3. Mtl1, Mid2, and Wsc1 Are Required for Cyclin C Degradation in Response to Moderate Oxidative Stress

**Figure 3:**
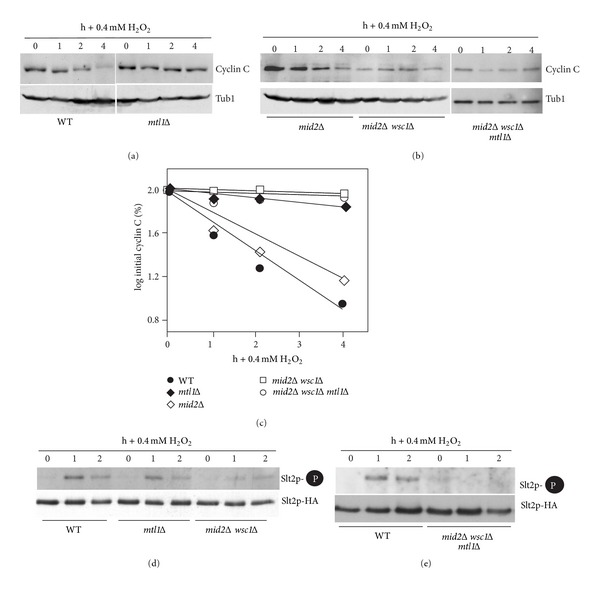
Mtl1 and Mid2/Wsc1 membrane sensors are required for cyclin C destruction following 0.4 mM H_2_O_2_ stress. (a) Wild type (RSY10) and *mtl1Δ* (RSY1660) expressing myc-cyclin C (pLR337) were grown to midlog phase (0 h) and then treated with 0.4 mM H_2_O_2_ for the indicated times in hours. Cyclin C levels were determined by Western blot analysis of immunoprecipitates. Tub1 levels were used as a loading control. (b) Cyclin C levels were monitored in *mid2Δ* (RSY1538), *mid2Δ  wsc1Δ* (RSY1547), and *mid2Δ wsc1Δ mtl1Δ* (RSY1707) strains as described in (a). (c) Quantification of the results obtained in (a) and (b). (d) T-loop phosphorylation (top panel) and immunoprecipitations (bottom panel) of Slt2-HA in wild type, *mtl1Δ, mid2Δ wsc1Δ,* and (e) *mid2Δ wsc1Δ mtl1Δ* following 0.4 mM H_2_O_2_ treatment for the times indicated (h).

*N*-Glycosylation is required for the proper function of Mid2, a cell wall sensor that is required to transduce the cell wall stress signal [13]. To determine which cell wall sensor (or sensors) is required to transmit the stress signal to cyclin C, we examined cyclin C levels in sensor mutants following exposure to H_2_O_2_ (0.4 mM). Cyclin C was protected from destruction in the *mtl1Δ* strain indicating that Mtl1 is required for this process (Figure 3(a), quantitated in Figure 3(c)). However, deleting *MID2* did not significantly alter cyclin C degradation kinetics (Figure 3(b)) suggesting that a functional specialization exists between these two members of the *N*-glucosamine modified family. Previous studies have shown that Mid2 and Wsc1 have redundant functions as oxidative stress sensors [2]. Consistent with this finding, cyclin C was not destroyed following H_2_O_2_ stress in *mid2Δ wsc1Δ* cells (Figure 2(b)) indicating that these proteins perform overlapping functions controlling cyclin C destruction. Interestingly, the prestress cyclin C levels were lower in the double mutant compared to the wild type. This observation may reflect the activation of another stress pathway that recognizes instability in the cell wall due to the loss of these sensors (reviewed in [11]) resulting in partial cyclin C destruction. A similar reduction in cyclin C levels was observed in unstressed *slt2Δ* mutants [7]. Taken together, these data suggest that two sensor groups, Mid2/Wsc1 and Mtl1, mediate H_2_O_2_-induced cyclin C destruction.

Previous studies indicated that the CWI MAPK Slt2 is required for H_2_O_2_ induced cyclin C destruction [7]. To determine if the Mid2/Wsc1 and Mtl1 sensor groups signal cyclin C destruction through Slt2, its activation was monitored by Thr and Tyr T-loop phosphorylation [51]. T-loop phosphorylation specific antibodies were used to probe Western blots of Slt2 immunoprecipitated from extracts prepared from wild type, *mtl1Δ*, or *mid2Δ wsc1Δ* strains before and after exposure to 0.4 mM H_2_O_2_. As previously reported [7] a transient elevation in phosphorylated Slt2 was detected in wild-type cells (Figure 3(d)). A similar result was obtained in the *mtl1Δ* strain while the *mid2Δ wsc1Δ* double mutant displayed a reduction in total Slt2 activation. To determine if both sensor groups contributed to Slt2 activation, the experiment was repeated in the *mid2Δ wsc1Δ mtl1Δ* triple mutant. This analysis revealed no detectable Slt2 phosphorylation under these conditions (Figure 3(e)). These results suggest that both Mid2/Wsc1 and Mtl1 groups are required for normal Slt2 activation in response to low-level H_2_O_2_ exposure.

### 3.4. Mtl1 Is *N*-Glycosylated on Asn42 in a Gfa1-Dependent Manner

**Figure 4:**
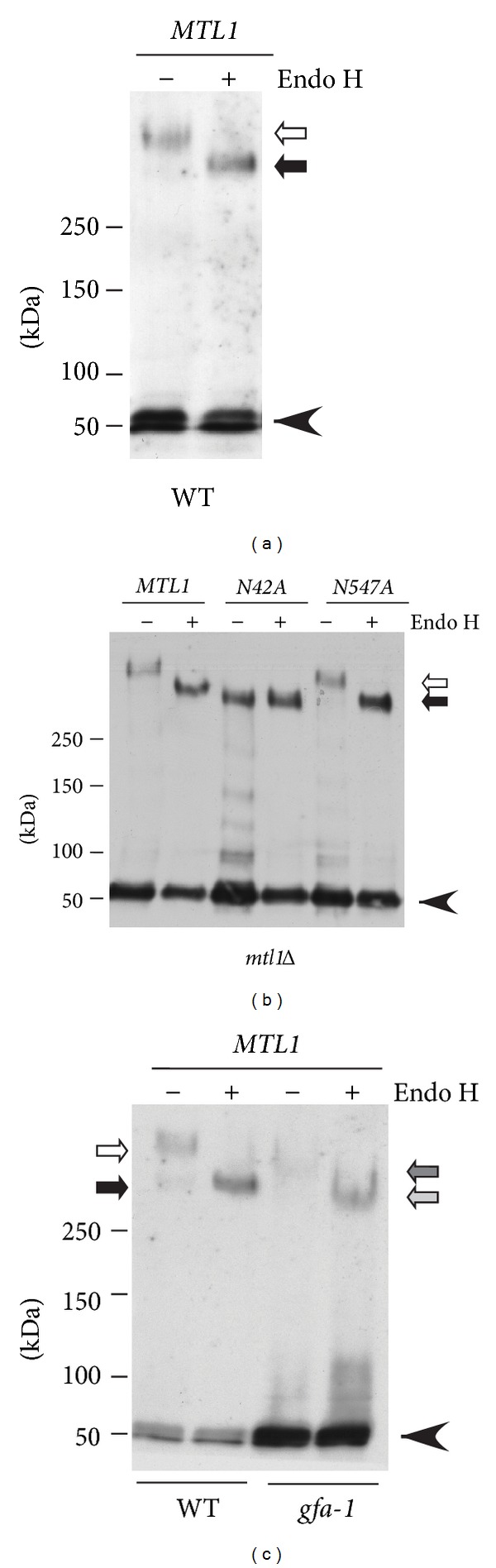
Mtl1 is glycosylated on Asn42. Immunoprecipitates from extracts prepared from a wild-type strain expressing Mtl1-3HA from its normal promoter were either subjected (+) or not (−) to Endo H treatment. Molecular weight standards (kDa) are given on the left. The arrowhead indicates the migration of the unmodified Mtl1-3HA. Open and closed arrows indicate modified Mtl1-3HA before and after Endo H treatment, respectively. (b) Site directed mutagenesis was used to substitute alanine for asparagine at position 42 or 547. The experiments described in (a) were repeated with the modified forms of Mtl1. (c) The experiment described in (a) was repeated with a wild type and *gfa1-1* mutant. Open and dark grey arrows indicated fully modified form in wild type and *gfa1-1* mutant, respectively. Closed and light grey arrows indicate the Endo H treated samples in wild type and the *gfa1-1* mutant, respectively.

Our results indicating that both Mtl1 and Gfa1 are required for ROS-induced cyclin C destruction prompted further experiments to determine if these proteins were functionally related. Given the role of Gfa1 in production of GlcNAc, we tested whether Mtl1 was *N*-glycosylated. The *MTL1* gene was tagged in the chromosome with three HA epitopes (3HA). Mtl1-3HA was immunoprecipitated and this immunoprecipitate was split and one half was treated with Endo H to remove glycosylation. Western blot analysis revealed that most of the Mtl1-3HA signal migrated at an apparent molecular weight of 56 kDa close to its predicted size (arrowhead, Figure 4(a)). A significantly slower species was also observed (open arrow) that shifted to a faster mobility upon Endo H treatment (solid arrow). The remaining high molecular weight Mtl1 species was most likely due to *O*-mannosylation. These results indicate that, as recently reported [52], Mtl1 is *N-*glycosylated. Inspection of the Mtl1 sequence for consensus *N*-glycosylation recognition sites (NxS/T, x*≠*P) revealed two motifs at Asn42 and Asn547. Site-directed mutagenesis was used to substitute alanines for these Asn residues and the experiments were repeated. Unlike the N547A mutant, Mtl1^N47A^ did not display the characteristic shift in mobility upon Endo H treatment (Figure 4(b)). These results indicate that Mtl1 is modified on N42. 

Finally, Mtl1 modification was examined in the *gfa1-1* mutant. In these experiments, two differences were observed compared to the wild-type control. First, Mtl1 mobility was enhanced following PAGE in the untreated mutant extract compared to wild type (Figure 4(c), compare open and dark grey arrows). Treatment of these samples with Endo H resulted in further mobility enhancement (compare solid and light grey arrows) although to a lesser extent. These results suggest that Mtl1 is still *N*-glycosylated to some extent, most likely due to residual Gfa1-1 activity. The second result was that the total amount of modified Mtl1 was reduced in the *gfa1-1* mutant with a corresponding increase in the unmodified form compared to wild-type extracts. In this experiment, twice the amount of the *gfa1-1* extract was required to visualize modified Mtl1. These results indicate that Mtl1 is *N*-glycosylated on Asn42 and that this modification requires Gfa1. In addition, the loss of Gfa1 activity reduces the steady state levels of modified Mtl1.

### 3.5. Mtl1 *N*-Glycosylation Is Required for Normal ROS-Induced PCD and Cyclin C Degradation

**Figure 5:**
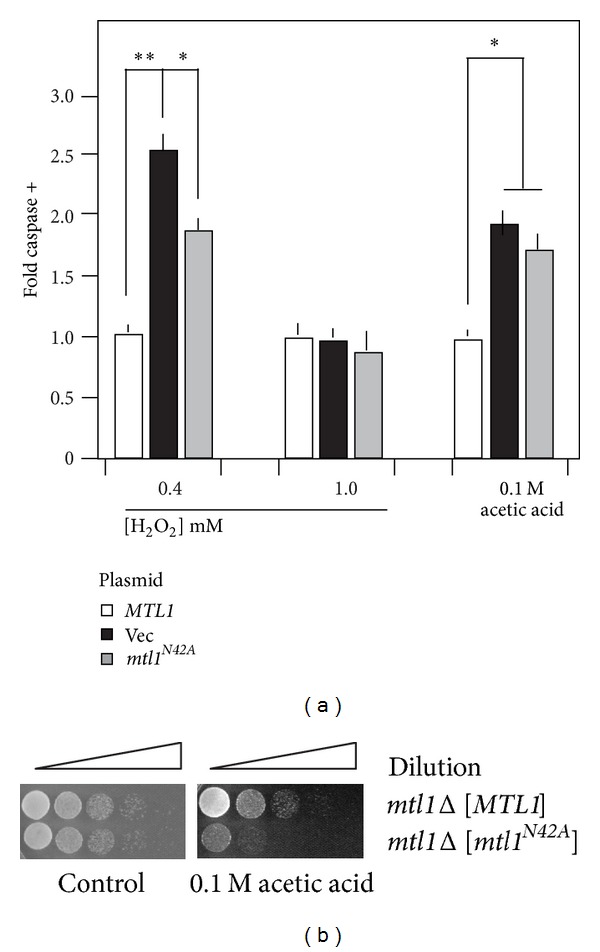
Mtl1 *N*-glycosylation is required for efficient stress-induced PCD execution. (a) The *mtl1Δ* strain (RSY1660) transformed with plasmids expressing *MTL1* (pCJ4), *MTL1* ^*N42A*^ (pCJ10), or the vector control was subjected to H_2_O_2_ or acetic acid at the indicated concentrations for 240, 200, and 200 m, respectively. Caspase assays were performed on three independent cultures and the mean ± S.E.M. is indicated. *P < 0.05, **P < 0.01. (b) Strains described in (a) were grown to midlog phase and then either treated or not with 100 mM acetic acid for 200 min. The cells were serially diluted (1 : 10) and then spotted onto growth medium. The images were obtained following 2 days incubation at 30°C.

Previous studies have found that mutants lacking Mtl1 are hypersensitive to H_2_O_2_ treatment [53]. To determine whether this loss in viability was due to PCD or necrosis, an *mtl1Δ* strain was transformed with plasmids expressing wild-type *MTL1* or the vector control. Three independent transformants were grown to midlog phase and subjected to 0.4 mM H_2_O_2_ for 4 h. These cells were analyzed for caspase activity using a fluorescent substrate and flow cytometry (see Section 2 for details). These studies revealed a significant increase in caspase active cells in the cells lacking *MTL1* expression (Figure 5(a)) indicating that at least a portion of the enhanced cell death observed in *mtl1Δ* mutants is due to PCD. To determine if *N*-glycosylation was required for this activity, the experiment was repeated with the *mtl1Δ* strain harboring a plasmid expressing *mtl1* ^*N42A*^. This study revealed an intermediate elevation in PCD compared to the wild type and the null strain values. These results suggest that loss of *N*-glycosylation reduces, but does not eliminate, Mtl1 function with respect to H_2_O_2_-induced PCD execution.

We next determined whether this intermediate phenotype was observed under two additional stress conditions known to induce PCD, namely, higher H_2_O_2_ concentrations or exposure to acetic acid. Surprisingly, exposing these cultures to higher H_2_O_2_ levels did not significantly change caspase activation between the three cultures. Conversely, the acetic acid treated *mtl1* ^*N42A*^ expressing strain exhibited similar levels of caspase activation compared to the vector control. This hypersensitivity to acetic acid was also observed in plating assays (Figure 5(b)) indicating that *N*-glycosylation is important for Mtl1 function following acetic acid treatment. These results indicate that Gfa1-dependent *N*-glycosylation of Mtl1 is required for the normal cellular response to low levels of H_2_O_2_ and acetic acid. However, this activity is dispensable in cells exposed to higher concentrations of peroxide. These results suggest that the cell can modulate the signal transduction configuration based on the extent and type of damage encountered.

### 3.6. Increasing H_2_O_2_ Concentrations Elevates CWI Pathway Activation

**Figure 6:**
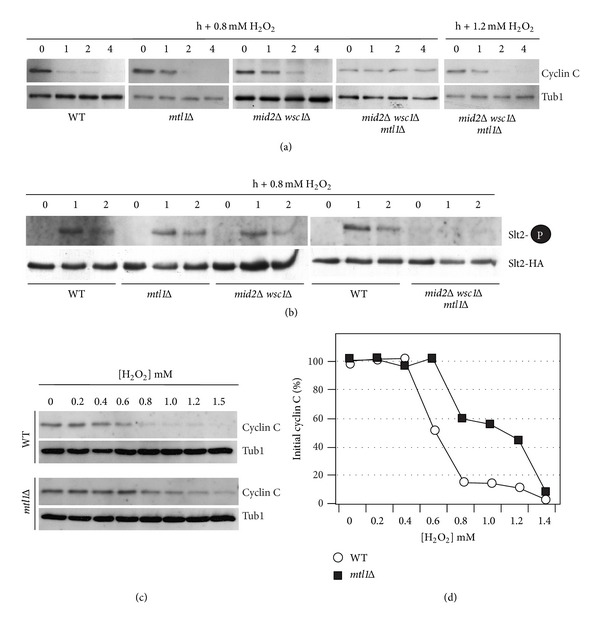
Membrane sensors are not required for cyclin C destruction following 0.8 mM H_2_O_2_ stress. (a) Wild type (RSY10), *mtl1Δ* (RSY1660), *mids2Δ wsc1Δ* (RSY1547), and *mid2Δ wsc1Δ mtl1Δ* (RSY1707) strains expressing myc-cyclin C (pLR337) were grown to midlog phase (0 hr) and then treated with 0.8 mM or 1.2 mM H_2_O_2_ for the indicated times. Cyclin C levels were determined by Western blot analysis of immunoprecipitates. Tub1 levels were used as a loading control. (b) Phosphorylation (top panels) and immunoprecipitation (bottom panels) of Slt2-HA in wild type, *mtl1Δ*, *mids2Δ wsc1*Δ, and *mid2Δ wsc1Δ mtl1Δ* strains following 0.8 mM H_2_O_2_ treatment. (c) Kinetics of cyclin C degradation are dependent upon the H_2_O_2_ dose. Wild type (RSY10) and *mtl1Δ* (RSY1600) strains expressing myc-cyclin C (pLR337) were grown to midlog phase (0 hr) and then treated with increasing amounts of H_2_O_2_ for 1 hour. Cyclin C levels were determined by Western blot analysis of immunoprecipitates. Tub1 levels were used as a loading control. (d) Quantification of cyclin C levels in (c).

The results just described indicated that Mtl1 is important for transducing a low-level ROS signal but not that of a higher concentration. To examine this question further, we investigated the role of the Mtl1 and Mid2-Wsc1 sensor groups in transducing high-level H_2_O_2_ exposure. Western blot analysis was used to monitor cyclin C levels before and following the addition of 0.8 mM H_2_O_2_ to wild type, *mtl1Δ*, *mid2Δ wsc1*Δ, and *mtl1Δ mid2Δ wsc1Δ* midlog-phase cultures. Unlike the results obtained with lower H_2_O_2_ concentration, loss of either the Mtl1 or Mid2/Wsc1 groups did not protect cyclin C from destruction (Figure 6(a)). Consistent with this finding, Slt2 activation was readily observed in both *mtl1Δ* and *mid2Δ wsc1Δ* mutants (Figure 6(b)). However, eliminating both groups (*mtl1Δ mid2Δ wsc1Δ*) stabilized cyclin C and suppressed Slt2 activation. These results suggest that as ROS exposure increases, either Mtl1 or Mid2/Wsc1 can initiate a signal sufficient to trigger cyclin C destruction. However, cyclin C destruction still occurs in the triple sensor mutant when the H_2_O_2_ concentration is raised to 1.2 mM (Figure 6(a)). These findings suggest that an additional pathway(s) is now enabled that can initiate cyclin C destruction under severe stress exposure (see Section 4). 

The above results suggest a model in which H_2_O_2_-mediated cyclin C destruction is regulated differently depending on the ROS dose. Two models seem most likely to describe these results. First, there may be a linear relationship between the amount of ROS applied and the effective rate for cyclin C destruction. Alternatively, there may be separate pathways whose activation occurs under specified stress conditions. To begin testing these possibilities, cyclin C levels were monitored in a wild-type strain exposed to increasing concentrations of H_2_O_2_. For these experiments, a single poststress timepoint of one hour was chosen as it represents an intermediate time so that changes in destruction kinetics can be identified. In wild-type cells, the addition of H_2_O_2_ up to 0.4 mM does not induce an appreciable decline in cyclin C levels after 1 h (Figure 6(c), quantitated Figure 6(d)). However, a significant reduction in cyclin C levels is observed at 0.6 mM H_2_O_2_. A continued reduction in cyclin C levels is observed at 0.8 mM but they remain relatively constant through 1.2 mM. These results are more consistent with the second model that additional pathways able to trigger cyclin C destruction are enabled in the 0.8–1.2 mM H_2_O_2_ concentrations. To further test this model, the experiment was repeated with the *mtl1Δ* mutant. Interestingly, following a delay in cyclin C destruction until 0.6 mM, a very similar profile was observed except that the 0.8 to 1.2 mM plateau occurred around 50% of cyclin C remaining versus approximately 15% for the wild-type strain. These results suggest that Mtl1 contributes to the oxidative stress signal regardless of the level of ROS and that additional pathways are activated to destroy cyclin C as ROS exposure increases (see Section 4).

### 3.7. Sensor Concentration Regulates the Cyclin C Destruction Signal

**Figure 7:**
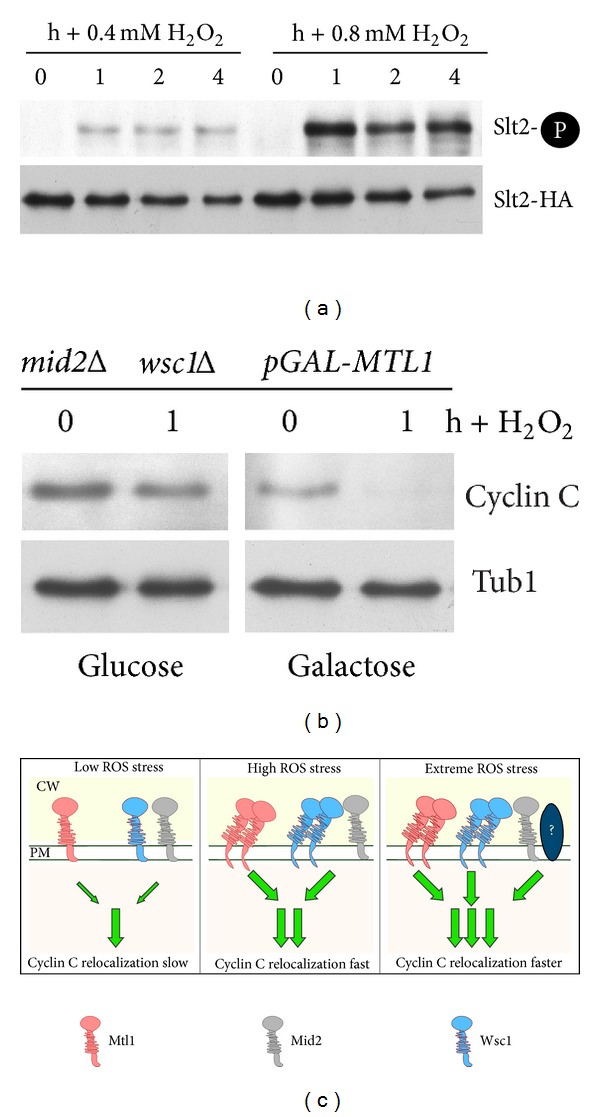
Mtl1 overexpression suppresses *mid2Δ wsc1Δ* signaling defect. (a) Phosphorylation (top panels) and immunoprecipitation (bottom panels) of Slt2-HA in wild-type strain (RSY10) following 0.4 mM and 0.8 mM H_2_O_2_ treatment as indicated. (b) A *mid2Δ wsc1Δ* mutant (RSY1844) harboring an integrated copy of *MTL1* under the control of the *GAL1* promoter and cyclin C-myc (pLR337) was grown to midlog in raffinose. The culture was split and treated with either 2% galactose or glucose for 2 h before the addition of 0.4 mM H_2_O_2_. Cyclin C levels were monitored by Western blot analysis of immunoprecipitates. Tub1 levels were used as a loading control. (c) Model of combinatorial signal transduction pathway control for cyclin C regulation. Sensors associated with the plasma membrane (PM) and cell wall (CW) are indicated. Sensor activity in response to low, high, and extreme ROS exposure is indicated. Under low stress conditions, the combined signals from both Mtl1 and Mid2/Wsc1 are required to trigger cyclin C relocalization and destruction. High stress conditions generate more signal from each group, perhaps through sensor clustering as proposed for Wsc1 [12, 54]. Extreme stress environment proposes the existence of another pathway, represented here by the question mark, that contributes to the overall stress. The location of this pathway at the plasma membrane was done for convenience and does not imply another cell wall sensor system per se.

The results just described suggest a model that increased oxidative stress is able to induce more rapid destruction of cyclin C. To determine if this difference in destruction kinetics could be explained by elevated Slt2 activation, the extent of T-loop phosphorylation was monitored in cultures exposed to low (0.4 mM) or high (0.8 mM) H_2_O_2_ concentrations. These concentrations were chosen as they represent the two steps observed with cyclin C destruction (see Figure 6(d)). These experiments revealed that Slt2 activation was elevated in cultures subjected to 0.8 mM H_2_O_2_ compared to 0.4 mM (Figure 7(a)). In addition, cyclin C destruction kinetics are enhanced in response to high ROS conditions (compare cyclin C degradation kinetics between Figures 3(a) and 6(a)). Taken together, these results suggest that activation of multiple stress pathways leads to heightened Slt2 activation and more rapid cyclin C destruction.

Since elevated H_2_O_2_ concentrations are able to increase Slt2 stimulation, we next asked if the different stress sensors generated a specific signal or Slt2 was simply responding to overall signal intensity. To test these possibilities, a plasmid containing *MTL1* under the control of the galactose inducible promoter (*GAL1*) was introduced into a *mid2Δ wsc1Δ* double mutant. In the presence of the repressing carbon source glucose, cyclin C was not destroyed in response to low H_2_O_2_ levels in the double mutant (Figure 7(b)) as we observed previously (see Figure 3(b)). Growing this culture in galactose medium, which induced Mtl1 overexpression, allowed normal cyclin C destruction to occur. These results indicate that overexpression of Mtl1 can compensate for the loss of the Mid2/Wsc1 signal. This finding is consistent with a model that overall sensor activity, and not a sensor-specific signal, mediates cyclin C destruction. Taken together, these results indicate that elevated sensor signaling, either by increasing the sensor or H_2_O_2_ concentrations, can more efficiently induce cyclin C destruction.

## 4. Discussion

ROS generated by oxidative phosphorylation in the mitochondria are normally neutralized by the intrinsic anti-oxidant system. The addition of exogenous prooxidants (peroxides, heavy metals) triggers the oxidative stress response. Low-level oxidative stress induces cell cycle arrest until the damage has been repaired. Conversely, extensive ROS-induced damage activates the programmed cell death pathway. Our previous studies found that oxidative stress induces the nuclear to cytoplasmic relocalization and destruction of the stress response gene transcriptional repressor cyclin C. In addition, cyclin C translocation is required for efficient PCD execution making the signaling pathway that transmits the ROS signal critical for the decision to enter PCD. In this report, we describe the sensor array that transmits the cyclin C destruction signal following H_2_O_2_ treatment. Using cyclin C relocalization and destruction as readouts, this study revealed that the cell wall sensors Mtl1 and either Mid2 or Wsc1 combinations are required for low-level ROS signaling to cyclin C. However, our data suggest that the cell is sensing the total signal, not a specific activity derived from each of these sensors. In addition, Mtl1 signaling requires *N*-glycosylated via a Gfa1-dependent process. In response to higher concentrations of prooxidant, additional pathways are activated that do not require these sensors. These findings indicate that the cell is able to respond to differing levels of oxidative stress through activation of multiple signaling pathways.

This study was initiated by the finding that a mutation in *GFA1* caused cells deleted for cyclin C to grow very slowly. This synthetic growth defect suggested that these factors somehow functioned in similar or redundant pathways. However, our data indicate that Gfa1 is required for the ROS-induced destruction of cyclin C indicating opposing functions. Thus, why are *cnc1Δ gfa1-1* mutants synthetically sick? One possibility takes into consideration that cyclin C is part of the Cdk8 module that represses over 100 genes [55, 56] while Gfa1 controls the modification of proteins through the production of GlcNAc and GPI anchors. Therefore, there are multiple opportunities for these two mutations to interact. For example, cyclin C-Cdk8 represses the transcription of *GIP2* [55], an activator of the protein phosphatase Glc7 [57]. Hyperactivation of Glc7 displays a growth defect in combination with mutations in the CWI pathway [58], which requires glycosylated Mlt1 for normal activity. Therefore, although the interactions may not be direct, the proper function of both cyclin C and Gfa1 as regulators of the stress response is important for normal cell growth.

An important question is how the cell responds to differences in stress signal intensities. The Wsc group is proposed to function as a mechanosensor in which the transmembrane domain anchors the protein in the plasma membrane while the head groups provide a dynamic interaction with the cell wall [24, 54, 59]. Here, we have identified specific sensor combinations that are required for cyclin C destruction. A current model in the field suggests that sensor concentration is an important factor for relaying the intensity of the damage signal [54, 59]. Our results can also be viewed in this overall framework. At low ROS levels, the combined activity of the Mtl1 and Wsc1/Mid2 sensor array is necessary to generate a signal sufficient to induce cyclin C destruction (Figure 7(c)). In response to elevated oxidative stress, each sensor group generates a signal on its own sufficient to induce cyclin C translocation to the cytoplasm. This ability may be partially explained by a clustering mechanism just described. At high stress levels, inactivating either Mtl1 or Mid2/Wsc1 still permits the generation of a signal sufficient to induce cyclin C relocalization and destruction. Our finding that simply increasing the levels of Mtl1 can suppress the requirement for Mid2 and Wsc1 suggests that overall signal intensity, not a specific signal generated by each senor type, is being read by the cell. By sensing the increased overall signal, the cell could respond by a more intense and sustained Slt2 activation, which in turn promotes a more rapid translocation of cyclin C to the cytoplasm. Thus, the rate of cyclin C appearance in the cytoplasm may represent a measure for the extent of the cellular damage encountered. 

Since cyclin C is still destroyed in the absence of Mtl1 and Mid2/Wsc1 at extreme stress levels, this model would require that an additional, yet undefined, pathway (or pathways) activated is able to contribute cyclin C translocation. One possible pathway would involve the additional Wsc family members Wsc2 and Wsc3. Wsc2 overexpression is able to suppress the requirement for Mid2 [10] suggesting that a functional overlap exists between these sensors. An alternative, but not mutually exclusive, possibility is that as exogenously added H_2_O_2_ increases, lipids, protein, and finally DNA are oxidized [60]. Oxidized proteins are selectively degraded by the proteasome [61] while DNA damage is sensed by several well-characterized pathways [62]. Maintenance of the redox state in the ER is critical for proper disulfide bond formation by Ero1 during nascent peptide folding (see [63] for review). An oxidized ER environment restricts Ero1 function resulting in misfolded proteins and ultimately ER stress. In addition, a product of Ero1 function is H_2_O_2_, which can add to the oxidative stress load especially under condition of elevated protein synthesis and/or defects in the protein folding system. For example,* N*-glycan addition is important for the folding of secretory proteins. Inactivation of this *N*-glycosylation step through the use of tunicamycin can initiate ER stress which involves an ER to nuclear translocation of a transcription factor [64]. Interestingly, cyclin C is partially destroyed in cells treated with tunicamycin [65] although Slt2 was not activated. These observations suggest that ER stress induced by protein folding deficiencies can also trigger cyclin C destruction. Therefore, depending on the level of ROS exposure, the signaling repertoire of the cell is altered to reflect detected damage at different cellular compartments. By having these pathways conflating their respective signals at cyclin C, the cell could detect and respond to significant damage even if one pathway is defective.

## Figures and Tables

**Table 1:** Yeast strains used in this study.

Strain	Genotype	Source
RSY10^a^	*MA* *T * **a ** *ade2 ade6 can1-100 his3-11, 15 leu2-3, 112 trp1-1 ura3-1 *	[41]
W303-1B^b^	*MA* *T * **α** * ade2 can1-100 his3-11, 15 leu2-3, 112 trp1-1 ura3-1 *	[42]
RSY391^a^	*cnc1::LEU2 *	[25]
RSY397^ b^	*cnc1::LEU2 *	This study
RSY1543^ b^	*cnc1::LEU2 gfa1-1 *	This study
RSY1539^a^	*chs3::KANMX4 *	This study
RSY1538^b^	*mid2::HIS3 *	This study
RSY1547^a^	*wsc1::KANMX4 mid2::HIS3 *	This study
RSY1659^a^	*MTL1-3HA::HIS3 *	This study
RSY1608^a^	*cnc1::LEU2 mtl1::TRP1 *	This study
RSY1660^a^	*mtl1::HIS3 *	This study
RSY1661^b^	*cnc1::LEU2 gfa1-1 MTL1-3HA::TRP1 *	This study
RSY1707^b^	*wsc1::KANMX4 mid2::HIS3 mtl1::TRP1 *	This study
RSY1844^b^	*wsc1::KANMX4 mid2::HIS3 pGAL-MTL1 *	This study

^a^Genotype: *MAT* **a** *ade2 ade6 can1-100 his3-11, 15 leu2-3, 112 trp1-1 ura3-1.*

^b^Genotype: *MAT* **α** *ade2 can1-100 his3-11, 15 leu2-3, 112 trp1-1 ura3-1*.

**Table 2:** Primers used in this study.

Name	Mutation created	Oligonucleotide
MID2 F1	*mid2*Δ	5′ TCGTTGAAGATTGGACATATAAAATACGCAAATCATAGTCGGATCCCCGGGTTAATTAA 3′
MID2 R1	*mid2*Δ	5′ AGGAATGAAAAGTAGCCATAAGCACTAAATGATATCAATGAATTCGAGCTCGTTTAAAC 3′
WSC1 F	*wsc1*Δ	5′ GTA AAC TCG ACC AGG CAC 3′
WSC1 R	*wsc1*Δ	5′ TATATCGTCTTTCAACGCGG 3′
MTL1 F1	*mtl1*Δ	5′ TTAACTTACTCCCAGTTAGTATAATATAAGTAGTTAAGGTCGGATCCCCGGGTTAATTAA 3′
MTL1 R1	*mtl1*Δ	5′ TTAAGAAGAAAAGTTATGGCAAAGCTGCTTTCGCTATGATGAATTCGAGCTCGTTTAAAC 3′
MTL1 F2	*MTL1-3HA *	5′ TATTACACGAAACCAAACAACGGCTTAAATATCACGAACTATCGGATCCCCGGGTTAATTAA 3′
MTL1 F4	*PGAL-MTL1 *	5′ TTTAAACACTTCTAGTTCATTTCGGGTTGGTTCGATCTTGGAATTCGAGCTCGTTTAAAC 3′
MTL1 R2	*PGAL-MTL1 *	5′ TTGAAGCAGAGCTCTTCTTCCTGGTCGGATTGCAGCTTGCCATTTTGAGATCCGGGTTTT 3′
N42A-F	Mtl1^N42A^	5′ GCAGGAGTTGGTTCCAGCTGCTAGCACAACATCGAGCAC 3′
N42A-R	Mtl1^N42A^	5′ GTGCTCGATGTTGTGCTAGCAGCTGGAACCAACTCCTGC 3′
N547A-F	Mtl1^N547A^	5′ GAAACCAAACAACGGCTTAGCTATCACGAACTATTAAATC 3′
N547A-R	Mtl1^N547A^	5′ GATTTAATAGTTCGTGATAGCTAAGCCGTTGTTTGGTTTC 3′
KCO1234	Amplify *gfa1-1 *	5′ CGCTTACAAGAAAGCATTC 3′
KCO1235	Amplify *gfa1-1 *	5′ GTCTAATTTAGGGCTGCAAC 3′

Oligonucleotides used in this study with their accompanying mutation identified.

**Table 3:** Plasmids used in this study.

Plasmid name	Gene	Epitope tag	Marker	Promotor	2*µ* or CEN	Reference
pYO964	*RHO1* ^ G19V^	No	*URA3 *	*ADH1 *	CEN	[66]
pKC311	*CNC1 *	No	*URA3 *	*CNC1 *	CEN	This study
pKC800	*GFA1* and *LAP4 *	No	*TRP1 *	*GFA1* and *LAP4 *	CEN	This study
pKC811	*LAP4 *	No	*TRP1 *	*LAP4 *	CEN	This study
pKC812	*GFA1 *	No	*TRP1 *	*GFA1 *	CEN	This study
pID301	*CNC1 *	No	*URA3* and *ADE2 *	*CNC1 *	CEN	This study
pBK37	*CNC1 *	YFP	*TRP1 *	*ADH1 *	CEN	This study
pLR106	*BCK1-20 *	No	*HIS3 *	*ADH1 *	CEN	[7]
pLR337	*CNC1 *	1 myc	*TRP1 *	*ADH1 *	CEN	[25]
MPK1-HA	*SLT2 *	HA	*LEU2 *	*MPK1 *	2*µ*	[67]
pCJ3	*MTL1 *	3HA	*HIS3 *	*ADH1 *	CEN	This study
pCJ4	*MTL1 *	3HA	*URA3 *	*ADH1 *	CEN	This study
pCJ11	*MTL1* ^ N42A^	3HA	*URA3 *	*ADH1 *	CEN	This study
pCJ12	*MTL1* ^ N42A^	3HA	*HIS3 *	*ADH1 *	CEN	This study
pCJ13	*MTL1* ^ N547A^	3HA	*HIS3 *	*ADH1 *	CEN	This study
